# iTRAQ-Based Quantitative Proteomics Analysis of the Protective Effect of Yinchenwuling Powder on Hyperlipidemic Rats

**DOI:** 10.1155/2017/3275096

**Published:** 2017-08-14

**Authors:** Zheyu Zhang, Wenbo Wang, Ling Jin, Xin Cao, Gonghui Jian, Ning Wu, Xia Xu, Ye Yao, Dongsheng Wang

**Affiliations:** Institute of Integrated Traditional Chinese and Western Medicine, Xiangya Hospital, Central South University, Changsha 410008, China

## Abstract

Yinchenwuling powder (YCL) is an effective traditional Chinese medicine formula to modulate lipid levels. In this study, we established hyperlipidemic rat models and treated them with YCL. The serum concentrations of lipid, malondialdehyde (MDA), endothelin-1 (ET-1), and calcitonin gene-related peptide (CGRP) were measured. Adventitia-free vascular proteins between hyperlipidemic rats and YCL-treated rats were identified using iTRAQ-based quantitative proteomics research approach. Proteins with 1.3-fold difference were analyzed through bioinformatics, and proteomic results were verified by Western blot. The results showed that the serum levels of TC, TG, LDL-C, ET-1, and MDA were significantly decreased, whereas the HDL-C and CGRP levels were significantly increased in the YCL-treated group. Proteomics technology identified 4,382 proteins, and 15 proteins were selected on the basis of their expression levels and bioinformatics. Of these proteins, 2 (Adipoq and Gsta1) were upregulated and 13 (C3, C4, C6, Cfh, Cfp, C8g, C8b, Lgals1, Fndc1, Fgb, Fgg, Kng1, and ApoH) were downregulated in the YCL-treated rats. Their functions were related to immunity, inflammation, coagulation and hemostasis, oxidation and antioxidation, and lipid metabolism and transport. The validated results of ApoH were consistent with the proteomics results. This study enhanced our understanding on the therapeutic effects and mechanism of YCL on hyperlipidemia.

## 1. Introduction

The prevalence rate of hyperlipidemia is increasing, and enhanced treatments for this conditions should be developed [1]. Multiple vascular disorders, including atherosclerosis, angiostenosis, and blocking, are the cause of aging and death in humans. Endothelial dysfunction represents abnormalities in early stages of vascular disease development, and this condition is pathophysiologically linked to subsequent atherosclerosis progression and cardiovascular disease events [2]. Impaired endothelial function has also been examined in animal models and healthy volunteers with sucrose and fat diet [3, 4]. Nevertheless, further studies should elucidate how various compounds act on vessel conditions and functions in hyperlipidemia.

Hyperlipidemia belongs to the category of “phlegm turbidity” in traditional Chinese medicine (TCM). In particular, shortage of spleen induces excess phlegm and moisture, which can cause thermogenesis. Therefore, studies on hyperlipidemia have focused on establishing the therapeutic values of traditional compounds to eliminate heat and promote diuresis. For example, Yinchenwuling powder (YCL) is a traditional Chinese medicinal formula consisting of six herbal materials, namely,* Artemisia capillaris* Thunb,* Alisma plantago-aquatica*,* Poria cocos*,* Polyporus umbellatus*,* Atractylodes macrocephala*, and* Cinnamomum cassia*. The earliest record of YCL can be found in* Synopsis of the Golden Chamber*, which is a classical Chinese ancient book in the Eastern Han Dynasty in the third century. In terms of the compatibility of herbs,* A. capillaris* Thunb is considered the major component because of its ability to eradicate “damp heat.” Wuling powder, comprising* A. plantago-aquatica*,* P. cocos*,* P. umbellatus*, and* A. macrocephala*, induces diuresis, dispels dampness, and invigorates the spleen.* C. cassia* favors the yang, promotes the circulation of qi, and relieves water retention. YCL also effectively regulates blood lipid metabolism [5–7]. Further investigations have confirmed that YCL alleviates lipid peroxide in hyperlipidemic rats, improves insulin resistance, and decreases serum inflammatory cytokines in hypertriglyceridemia patients [8, 9]. YCL also enhances the main hemorheological index in atherosclerotic rats by reducing plasma viscosity and platelet adhesion ratio [10]. Target proteins associated with effective YCL treatment on hyperlipidemia have also been determined [11].

In this study, the protective effects of YCL on vascular endothelial functions in hyperlipidemic rats were revealed through molecular biological techniques. Isobaric tags for relative and absolute quantification were also utilized on the basis of proteomic technology to identify differentially expressed proteins in adventitia-free aorta between experimental hyperlipidemic rats and YCL-treated rats. Our study enhanced our understanding on the therapeutic effects and mechanism of YCL on hyperlipidemia. 

## 2. Materials and Methods

### 2.1. YCL Powder Extraction

YCL was prepared with the following components: 30 g of* A. capillaris* Thunb, 15 g of* A. plantago-aquatica*, 9 g of* P. cocos*, 9 g of* P. umbellatus*, 9 g of* A. macrocephala*, and 6 g of* C. cassia*. These herbals were purchased from the Dispensary of Traditional Chinese Medicine, Xiangya Hospital, Central South University (Changsha, China). All of these components were identified by Xinzhong Li. The YCL extracts were obtained as follows.* A. capillaris* Thunb (300 g),* A. plantago-aquatica* (150 g),* P. cocos* (90 g),* P. umbellatus* (90 g),* A. macrocephala* (90 g), and* C. cassia* 60 g were weighed and added in 10-fold water twice once per hour. The mixed decoction was concentrated to 0.8 g/ml, filtered with a filter paper to separate all kinds of solid particles, and placed in a refrigerator at 4°C until use.

As the control group, Xuezhikang capsule (XZK; Peking University WBL Biotech Co., Ltd., China) was dissolved in double distilled water and then concentrated to 0.1 g/ml.

### 2.2. Animal Studies

Sprague–Dawley male rats (180–220 g, *n* = 40) were obtained from the Laboratory Animal Center of Central South University. They were kept under controlled relative humidity (50%–55%) and room temperature (18°C–26°C) with a 12 h/12 h light/dark cycle. Food and water were accessible to the rats. All of the experimental procedures and protocols were approved by the Animal Ethics Committee of Central South University, China.

The rats were adaptively fed for 1 week and then divided into four groups: The control group (*n* = 10) received basic forage. The model group or the hyperlipidemic (*n* = 10) group was provided with a high-fat diet. The YCL group (*n* = 10) was given a high-fat diet and treated with YCL extract. The XZK group (*n* = 10) was subjected to a high-fat diet and treated with XZK capsule solvent.

The control group was fed with normal pellet diet. The model, YCL, and XZK group were given a modified high-fat diet. The medical intervention was administered to all of the rats starting from the fifth week. The rats from the control group and the model group were given double distilled water. The rats from the YCL group received 0.8 g/ml YCL extracts. The rats from the XZK group were treated with 0.1 g/ml XZK capsule solution. Additionally, 2.5 ml of the liquid was given twice daily for 4 weeks.

After the experiment was completed, all of the rats were anesthetized by intraperitoneally injecting 10% chloral hydrate (0.35 ml/100 g). Blood sampled from the jugular vein was centrifuged at 2000 ×g for 20 min. A part of the collected serum was used to detect lipid levels, and the remaining part was stored at −20°C for subsequent analysis. Vascular tissues were derived from the thoracic aorta after the rats were dissected. Fat, blood, and connective tissues were initially removed from the vascular surface in cold saline. The gross adventitial tissue was subsequently removed under a stereoscopic microscope, and the adventitia-free vascular tissue was stored at −80°C for proteomic analysis.

Serum TC, TG, HDL-C, and LDL-C concentrations were measured by using an automatic biochemical analyzer (AU680, BECKMAN COULTER).

Serum concentrations of malondialdehyde (MDA), endothelin-1 (ET-1), and calcitonin gene-related peptide (CGRP) were identified in the four groups of rats to evaluate vascular endothelial functions. The MDA (A003-1, 20160509, Jiancheng, Nanjing, China) concentrations were determined using an assay kit according to the manufacturer's instructions. The serum levels of ET-1 (W23030603, Huamei, Wuhan, China) and CGRP (V04030604, Huamei, Wuhan, China) were determined by using a selective ELISA kit according to the manufacturer's instructions.

### 2.3. Protein Extraction, Digestion, and iTRAQ Labeling

The samples were ground quickly until there were no obvious particles under liquid nitrogen conditions. Then, we added 800 *μ*l of RIPA lysate to the samples. The supernatant was centrifuged at 12,000 r/min for 20 min at 4°C for 1 min and then harvested. A BCA protein assay kit was used to determine the protein concentrations.

Filter-aided sample preparation was applied to digest the proteins [12–14]. Each step was described as follows. The protein (200 *μ*g) in each group was reduced by 4 *μ*L of reducing reagent (supplied by manufacturer) and incubated at 60°C for 1 h. The protein was mixed with 2 *μ*L of cysteine blocking reagent to react for 10 min at room temperature and treated with ultrafiltration. The samples were centrifuged at 12,000 ×g for 20 min, and the bottom solution of the collection tube was discarded. Afterward, 100 *μ*L of 1 M TEAB buffer was added and centrifuged at 12,000 ×g for 20 min under the same conditions. The bottom solution of the collection tube was discarded. After three replicates of this procedure were prepared, two different concentrations of trypsin were successively added to the proteins in a renewed tube with an interval of 2 h. The protein was 100 times the trypsin formerly and 50 times latterly. Subsequently, 1 M TEAB was added to adjust the volume of the samples to 50 *μ*L, and the samples were incubated overnight at 37°C. On the next day, the samples were centrifuged at 12,000 ×g for 20 min, and the bottom of the tube was collected. Afterward, 50 *μ*L of 1 M TEAB was added and centrifuged simultaneously under the same conditions. Merged with the last step, 100 *μ*L of the samples was obtained at the bottom of the collecting tube. The hyperlipidemic and model groups were labeled with iTRAQ tag 113 and tag 116 and, respectively, named group A1 and group A2. The YCL powder groups were labeled with tag 119 and tag 115 and, respectively, named group B1 and group B2. Vacuum centrifugation was used to dry the labeled peptide. All operation procedures were performed in accordance with the manufacturers' instructions.

### 2.4. High-pH Reverse-Phase Fractionation

High-pH reverse-phase fractionation chromatography (hp RP) was carried out on SHIMADZU LC-20AD. The labeled peptides were redissolved with mobile phase A and then placed into the chromatographic column (Gemini-NX 3u C18 110A; 150*∗*2.00 mm). Then, we separated the peptides with a linear gradient, which was prepared with mobile phase A (20 mM HCOONH_4_, pH 10) and mobile phase B (20 mM HCOONH_4_, 80% ACN). In this gradient, mobile phase B was from 5% to 40% in 30 min at a flow rate of 200 *μ*l/min, and the UV detector was set at 214 nm/280 nm. The fractions were collected with a frequency of one tube per minute in 24 EP tubes. Lastly, 24 collected fractions were mixed with 50% TFA and dried in a vacuum centrifuge for the next analysis.

### 2.5. Nano-LC-MS/MS Analysis by Q Exactive

LC-MS analysis was initially conducted on a chromatographic column (75 um i.d. × 150 mm, packed with Acclaim PepM-ap RSLC C18, 2 um, 100 Å, nanoViper). We separated the peptides with a linear gradient, which was obtained with mobile phase A (5% ACN, 0.1% FA) and mobile phase B (80% ACN, 0.1% FA). In this gradient, mobile phase B was from 5% to 90% for 50 min at a flow rate of 200 *μ*l/min and maintained at 4% in the minutes 50.2 and 60. MS/MS analysis was performed using a mass spectrometer (Thermo Scientific Q Exactive). The following MS parameters were set: mass range,* m*/*z *350–1800; resolution, 70,000; AGC target, 3e6; and maximum IT, 40 ms. The following parameters of the tandem mass spectra were used: resolution, 17, 500; AGC target, 1e5; maximum IT, 60 ms; TopN, 20; and NCE/stepped NCE, 30.

### 2.6. Protein Identification and Data Analysis

Proteins were identified using ProteinPilot™ 5.0 (AB Sciex). Our study retrieved the MS/MS spectra from RAT_uniprot_2015.11.28, which covered 29,982 of protein sequences. According to the standard parameters, a unique protein must contain at least two unique peptides, and the false discovery rate (FDR) applied to identify peptides and proteins was less than <1%. To strengthen credibility, we only considered the iTRAQ ratio of the proteins within 0.5–20. The confidence of the identified proteins must be more than 95%, and the protein confidence threshold cutoff was set to 1.3 (unused) with at least more than one peptide above the 95% confidence level. To designate the significant changes in protein expression, the cutoff values of fold changes were more than 1.3 or less than 0.769.

### 2.7. Bioinformatics

We further investigated the differentially expressed proteins with gene ontology (GO) analysis. The GO project consisted of three components: biological process, cellular component, and molecular function. Then, Kyoto Encyclopedia of Genes and Genomes (KEGG, http://www.genome.jp/kegg/kegg1.html) database was applied carried to show the significantly enriched pathway. Additionally, we adopted the STRING database to search the interaction network and functional relations in differentially expressed proteins.

### 2.8. Western Blot Analysis

Western blot was applied to confirm the differential expression of significantly changed proteins in our proteomic study via a standard experimental method. The proteins were separated on a 12% SDS-PAGE gel and transferred to polyvinylidene difluoride (PVDF) membranes (0.4 um, Millipore), and these membranes were immunoblotted with apolipoprotein H. IgG goat anti-rabbit antibodies conjugated with HRP were used as secondary antibodies (1 : 5000, BOSTER), and the PVDF membranes were visualized through enhanced chemiluminescence (ECL).

### 2.9. Statistical Analysis

Data were statistically analyzed in SPSS version 20.0 (SPSS, Inc., Chicago, IL, USA). Results were expressed as mean ± SD. One-way ANOVA and *T* test were conducted to determine significant differences. *P* < 0.05 was selected to indicate significant differences.

## 3. Result

### 3.1. Effect of YCL on Serum Lipid Levels

After the treatment was completed, the lipid levels in the serum were observed. The TG, TC, and LDL-C levels of the YCL-treated rats were remarkably lower and their HDL-C levels were higher than those of the hyperlipidemic rats (Table 1). The YCL-treated rats were different from the XZK-treated rats, but they did not significantly vary from each other.

### 3.2. Levels of MDA, ET-1, and CGRP

The molecular biology detection results showed that YCL increased the concentrations of CGRP, and their level in the YCL-treated groups was significantly different from that in the model groups (*P* < 0.05). YCL decreased the MDA and ET-1 levels, which significantly differed from those in the model groups (*P* < 0.05; Table 2).

### 3.3. Primary Data Analysis and Protein Identification

**Figure 1:**
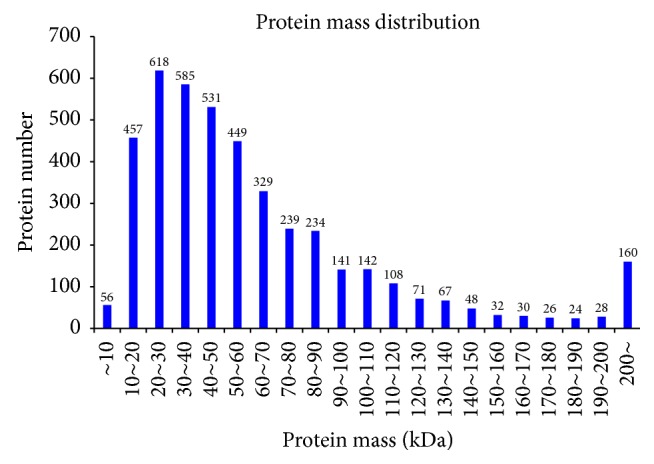
Protein mass distribution. Abscissa is the protein mass (kDa); ordinate is the protein number.

**Figure 2:**
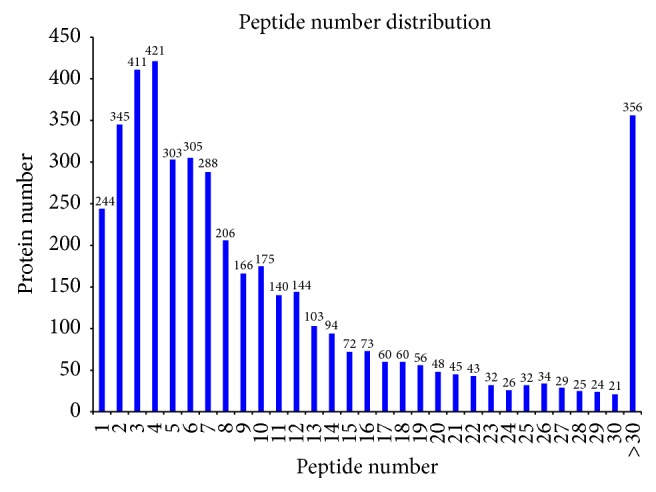
Distribution of peptide number. Abscissa denotes the peptide number. Ordinate corresponds to the protein number.

**Figure 3:**
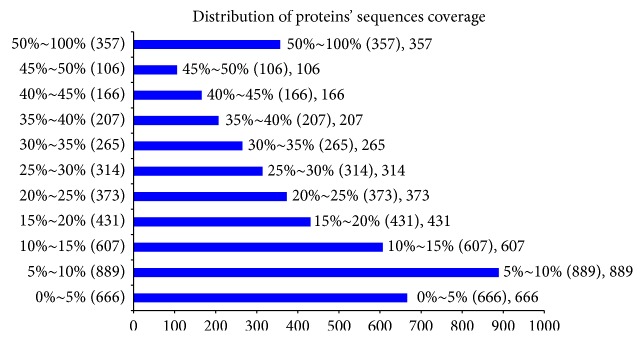
Distribution of the coverage of protein sequences. Abscissa denotes the proteins' sequences coverage and ordinate indicates the distribution.

**Figure 4:**
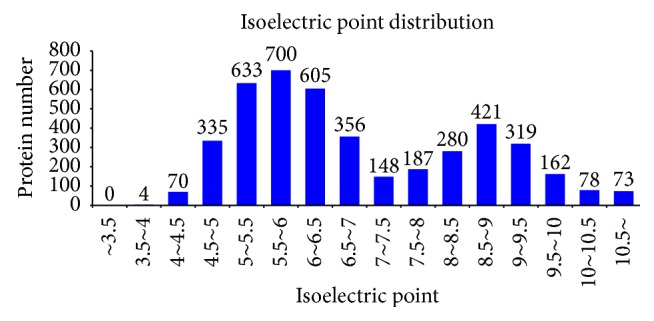
Distribution of isoelectric point. Abscissa denotes the isoelectric point and ordinate indicates the protein number.

**Figure 5:**
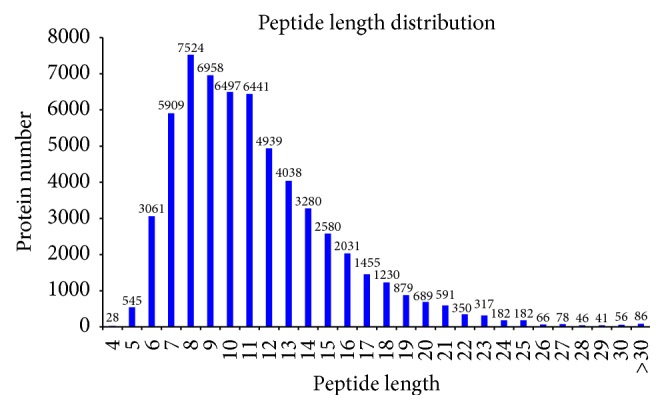
Distribution of peptide length. Abscissa denotes the peptide length and ordinate indicates the protein number.

In this experiment, iTRAQ was applied to identify the proteome changes between the model group and the YCL-treated group. A total of 54% spectra and 129,674 spectra were identified, and 66,653 distinct peptides, 18,593 proteins before grouping, and 4,382 proteins (at least two peptides with high confidence) were obtained. We observed 2,191, 1,392, 436, 140, and 160 proteins with a mass of 10–50, 50–100, 100–150, 150–200, and more than 200 kDa, respectively (Figure 1). The distribution of the peptide number is shown in Figure 2. The specific peptide number with 1, 2–5, 6–10, 11–20, 21–30, and above 30 peptides consisted of 244, 1,480, 1,140, 850, 311, and 356, respectively. The protein sequence coverage with 5%–10%, 40%–50%, 30%–40%, 20%–30%, 10%–20%, and below 10% variation accounted for 357 (8%), 272 (6%), 472 (11%), 687 (16%), 1,038 (24%), and 1,555 (35%), respectively (Figure 3). The distribution of isoelectric point is presented in Figure 4. Our results revealed 0, 74, 968, 1,305, 504, 467, 740, 240, and 73 proteins with isoelectric point below 3.5, 3.5–4.5, 4.5–5.5, 5.5–6.5, 6.5–7.5, 7.5–8.5, 8.5–9.5, and 9.5–10.5 and more than 10.5. Peptide length distribution below 5, 5–10, 10–15, 15–20, 20–25, and 25–30 and above 30 accounted for 573, 29,949, 21,278, 6,284, 1,622, 287, and 86, respectively (Figure 5).

### 3.4. A Total of 4,382 Proteins Were Altered within Adventitia-Free Aorta of the Experimental Hyperlipidemic Rats and the YCL-Treated Rats

**Figure 6:**
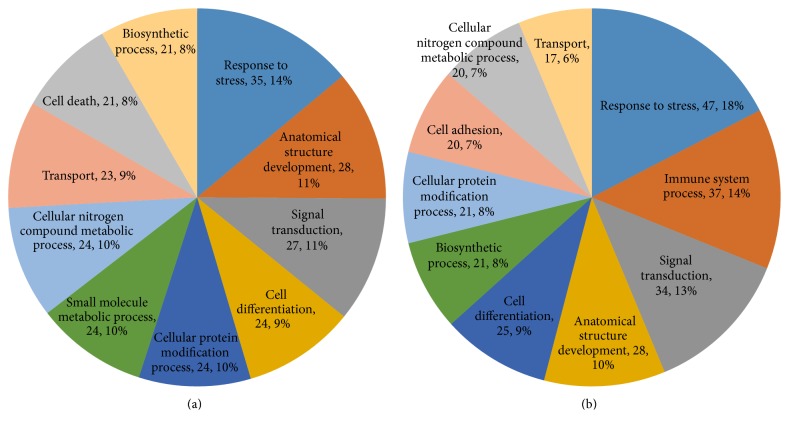
Biological process in GO assignment of differentially expressed proteins. (a) Upregulated proteins: the response to stress (14%) was the dominant feature. (b) Downregulated proteins: the response to stress (18%) was the major component.

**Figure 7:**
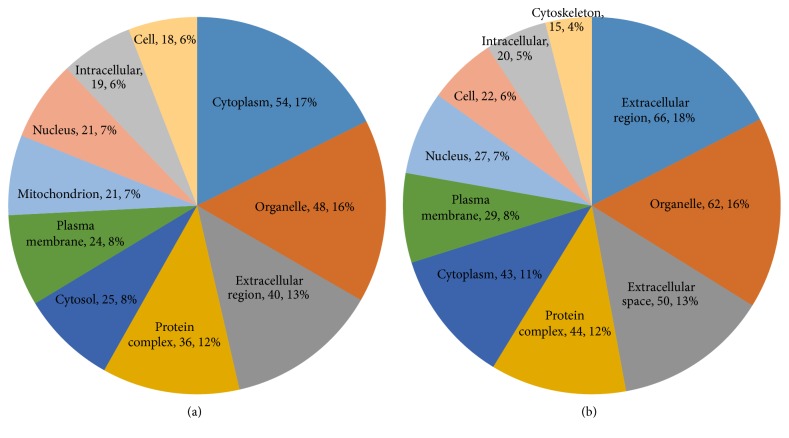
Cellular components in GO assignment of differentially expressed proteins. (a) Upregulated proteins: cytoplasm (17%) was the major component. (b) Downregulated proteins: extracellular region (18%) formed the main component of the cellular component category.

**Figure 8:**
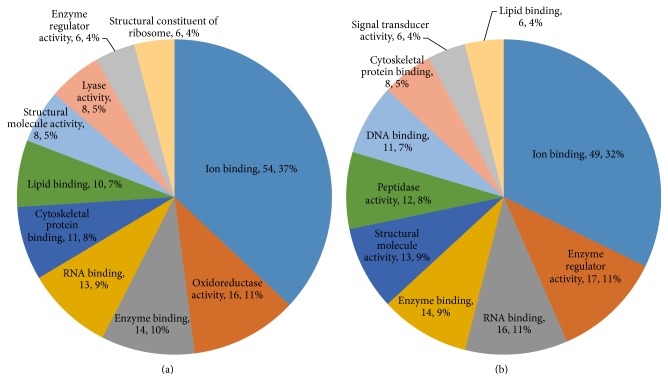
Molecular function in GO assignment of differentially expressed proteins. (a) Upregulated proteins: ion binding represented 37% of the molecular function. (b) Downregulated proteins: ion binding (32%) was the dominant molecular function in the GO assignments.

After we analyzed the protein profile, we found 4,382 differentially expressed proteins (*P* ≤ 0.05) with a FDR of less than 1%. Compared with those in the model group, 119 proteins in the YCL-treated group were upregulated more than 1.3-fold and 142 proteins were downregulated less than 0.769-fold. Supplementary Table 1 (in Supplementary Material available online at https://doi.org/10.1155/2017/3275096) describes the proteins in detail. GO analysis revealed a large amount of information. In terms of biological processes, 14% of the increased proteins were about “response to stress.” “Signal transduction” and “anatomical structure development” were followed equally, and each of these processes constituted 11% of the increased proteins (Figure 6(a)). Around 18% of the decreased proteins were about “response to stress” and “immune system process” and “signal transduction” ranked second and third with 14% and 11% of the increased proteins (Figure 6(b)). In terms of cellular components, 17% and 16% of the increased proteins were, respectively, related to the cytoplasm and the organelle, followed by extracellular region (13%; Figure 7(a)). Approximately 18% of the decreased proteins were linked to extracellular region and 16% of these proteins were attributed to organelle, followed by extracellular space (13%; Figure 7(b)). For molecular functions, 37% of the upregulated proteins were associated with ion binding, followed by oxidoreductase activity (16%; Figure 8(a)). Approximately 32% of the downregulated proteins were associated with ion binding, followed by 11% of these proteins related to enzyme regulation (Figure 8(b)).

These proteins were mapped to multiple pathways in the KEGG database to investigate the biological functions of these proteins (Table 3). “Systemic lupus erythematosus” was the most represented pathway, followed by “complement and coagulation cascades” and “metabolic pathways.” Those annotated protein species were significantly enriched in pathways of “alcoholism,” “viral carcinogenesis,” “*Staphylococcus aureus* infection,” “malaria,” “African trypanosomiasis,” and “platelet activation.”

**Figure 9:**
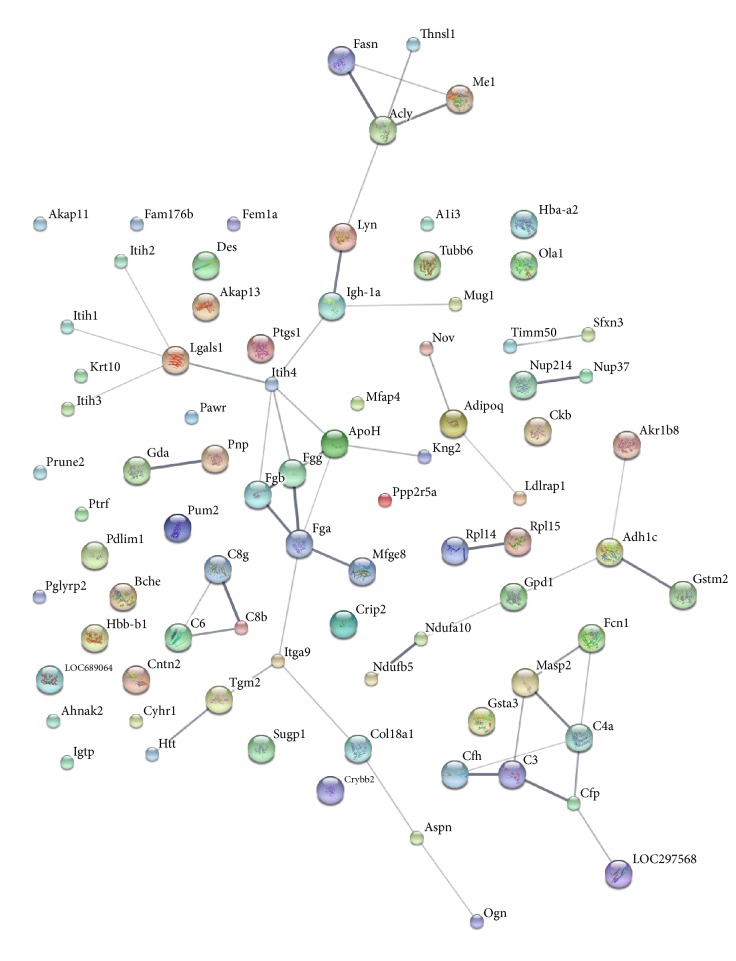
Interaction network analysis of differential expression of proteins. In this network, nodes denote proteins, lines represent functional associations between proteins, and line thickness corresponds to the level of confidence in the reported association.

The STRING tool was applied to present protein-protein interactions and their network (Figure 9). The interactions between proteins related to complement, immunity, inflammation, coagulation, and hemostasis were highly coexpressed. The proteins involved in immunity and inflammation were complement C3 precursor (C3), complement factor H precursor (Cfh), complement component 4, gene 2 precursor (C4a), properdin precursor (Cfp), ficolin-1 precursor (Fcn1), complement component 6 (C6), complement component C8 gamma chain (C8g), complement component C8 beta chain (C8b), and beta-2-glycoprotein 1 precursor (ApoH). Fibrinogen gamma chain (Fgg), fibrinogen beta chain (Fgb), fibrinogen alpha chain isoform 2 precursor (Fga), integrin alpha 9 (Itga9), protein-glutamine gamma-glutamyl transferase 2 (Tgm2), T-kininogen 2 (Kng2), and inter-alpha-inhibitor H4 heavy chain precursor (Itih4) were implicated in coagulation and hemostasis. The network shows that an interaction between 30 kDa adipocyte complement-related protein and protein Ldlrap1 existed in the upregulated proteins.

### 3.5. Validation of Differentially Expressed Proteins by Western Blot

**Figure 10:**
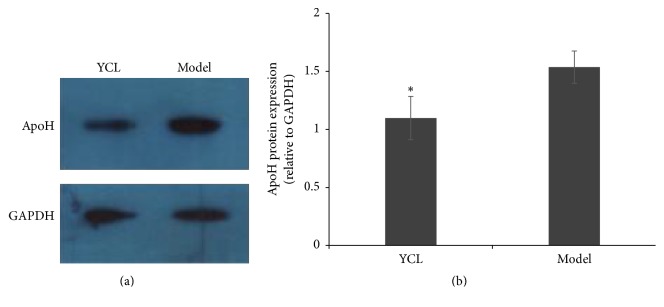
Confirmation of proteomics analysis results. The expression level of apolipoprotein H was measured by Western blotting (a). Quantitative analysis indicated that the expression of ApoH was significantly lower in the YCL-treated group than that of the model group (b). Data represent mean ± SD for *n* = 3. *∗* indicates significant difference as compared with the model group (*P* < 0.05).

In the section of protein validation, Western blot revealed the expression level of apolipoprotein H (Figure 10), and this result was consistent with the data of proteomics analysis. Compared with that of the model group, the expression level of apolipoprotein H was significantly decreased in the adventitia-free aorta of the YCL-treated group.

## 4. Discussion

YCL can effectively regulate blood lipid metabolism, alleviate lipid peroxide, improve insulin resistance, and decrease serum inflammatory cytokines [7–10]. The therapeutic targets, including T-kininogen, C3, C4, C4BPA, Ig*γ*-2 chain C, Mbl2, Hpx, FGL1, ApoE, ALB, TTR, and VDBP, of YCL in the plasma of hyperlipidemic individuals have also been identified through proteomic technology. These significant differentially expressed proteins modulated by YCL are into four aspects based on their functions: lipid metabolism and transport, coagulation and hemostatic processes, immunity and inflammation, and substance transport [11].

To create more specific targets, we focused on significant differentially expressed proteins selected on the basis of their expression levels and bioinformatics analysis. These differentially expressed proteins were significant with at least 1.5-fold changes. In Table 4, complement C3, complement C4, complement component C6, complement factor H precursor, properdin precursor, complement component C8 gamma chain, complement component C8 beta chain, adiponectin, galectins, fibronectin type III domain-containing protein 1, fibrinogen beta chain, fibrinogen gamma chain, kininogen-1, apolipoprotein H, and glutathione-S-transferase were included. These proteins are classified into several categories to recognize the potential therapeutically related drug targets of YCL in hyperlipidemic adventitia-free aorta.

### 4.1. Effects of YCL on Immune

Complement participates in the occurrence and development of atherosclerosis. The local expression levels of various complement components in advanced atherosclerotic plaques have been confirmed. The upregulation of complement components in the plasma and the aorta tissue is more prone to severe hypercholesterolemia [15]. The plasma concentrations of complement components increase in patients with coronary artery disease [16, 17]. Complement treatment as a target has been subjected to preliminary clinical trials on cardiovascular disease prevention [18]. Consistent with previous studies on plasma proteomes [11], our experiment confirmed that YCL treatment could decrease the expression of multiple complement components, including complement C3, complement C4, complement component C6, complement factor H precursor, properdin precursor, complement component C8 gamma chain, and complement component C8 beta chain. Therefore, YCL may contribute to early stages of hypercholesterolemia preceding atheroma formation by intervening with the complement system.

### 4.2. Effects of YCL on Lipid Metabolism and Transport

Apolipoprotein H (ApoH), also called beta-2-glycoprotein I, is mainly synthesized in the liver. ApoH activates lipoprotein lipase and participates in the metabolism of lipoproteins, particularly triglyceride-rich lipoproteins [19, 20]. A clinical research has found that the concentration of ApoH increases in patients with hyperlipidemia, and this observation suggests a close link between plasma lipoprotein and ApoH [21]. The pathogenesis of atherosclerosis is related to plasma lipoprotein metabolism, and ApoH is associated with the atherogenic and thrombotic processes [22]. ApoH may be proatherogenic by promoting an immune response in mice and likely increases the progression of atherosclerosis [23]. In our study, the expression of ApoH was significantly decreased in the YCL group compared with the hyperlipidemic group.

### 4.3. Effects of YCL on Inflammation

Adiponectin, an adipokine produced and secreted by adipose tissues, participates in energy metabolism, inflammation, and cell proliferation [24]. This adipokine exhibits multiple functions of antidiabetic, anti-inflammatory, antiatherogenic, and cardioprotective effects [25–27]. Adiponectin is implicated in lipid metabolism; this substance is negatively correlated with TG and LDL but is positively correlated with HDL [28]. In vivo studies have demonstrated that adiponectin supplementation can evidently reduce the proliferation of vascular smooth muscle cells and the thickening of the intima [29, 30]. Moreover, adiponectin protects the endothelium against hyperlipidemic injury via multiple mechanisms, such as promoting eNOS activity, inhibiting iNOS activity, preserving bioactive NO, and attenuating oxidative/nitrative stress [31]. Our study found that the increased expression of adiponectin was associated with the treatment of YCL.

Galectins are involved in intercellular signaling and cell-to-cell and cell-to-matrix adhesion, which may contribute to the pathogenesis of atherosclerosis [32]. Galectin-1, as a prominent GAL family member, plays an essential role in T-cell regulation [33, 34]. In connection with obesity, GAL1 is upregulated in the subcutaneous adipose tissues of obese patients and diet-induced obese mouse models [35, 36]. The upregulation of Gal-1 likely reinforces serum-induced events during vascular injury [37]. Our experimental results suggested that the level of galectin in the YCL group was lower than that in the hyperlipidemic group.

Fibronectin, a glycoprotein composed of repeating types I, II, and III modules, is synthesized by various cells. Fibronectin is closely involved as an proinflammatory factor in hyperlipidemia and atherosclerosis. Lipid concentrations may damage endothelial cells on blood vessel walls, and these mechanisms include fibronectin activation [38]. Fibronectin concentration is also positively correlated with the severity of atherosclerosis. Our study found that the decreased expression of fibronectin type III domain-containing protein 1 is associated with the treatment of YCL.

### 4.4. Effects of YCL on Coagulation and Hemostatic Processes

The fibrinogen molecule forms the substrate for thrombin and functions as an acute phase protein in the final step of the coagulation cascade. This molecule can modulate endothelial function, promote smooth muscle cell proliferation and migration, and interact with the binding of plasmin to its receptor [39]. Epidemiological follow-up studies have suggested that fibrinogen is a major primary cardiovascular risk factor and increased levels of fibrinogen and clotting activity are related to the rapid atherosclerosis progression [40]. Cross-sectional results have also demonstrated the associations between fibrinogen and cardiovascular risk factors or diseases [41]. The decreased expression of fibrinogen beta chain and fibrinogen gamma chain was linked to the treatment of YCL in our experiment, and this finding indicated that YCL could improve the function of blood coagulation in hyperlipidemia.

Kinin-kallikrein system is considered prothrombotic because it activates the intrinsic pathway and proinflammatory factors by producing the bioactive peptide bradykinin. Kininogen-1, as a constituent of the kinin-kallikrein system, is likely implicated in pathologic thrombus formation and inflammation [42]. Kininogen-1 can also promote the activation of the coagulation cascade by functioning as an auxiliary element of factor XI in blood coagulation [43]. High blood agglutination and hyperviscosity have been observed in hyperlipidemic rats [44]. Our experiment suggested that YCL treatment could downregulate the level of kininogen-1, which contributed to blood coagulation disorder in hyperlipidemia.

### 4.5. Effects of YCL on Oxidation and Antioxidation

Glutathione-S-transferase protects endothelial cells from damage induced by oxidants and toxins. Oxidative stress is detected in the pathogenesis of hyperlipidemia and atherosclerosis [45]. Glutathione-S-transferase activities significantly decrease in experimental rabbits fed with a hyperlipidemic diet compared with those of control rabbits provided with a standard diet [46]. In our experiment, the YCL treatment upregulated the expressed level of glutathione-S-transferase, which contributed to oxidative stress state in hyperlipidemia.

## 5. Conclusions

In this experiment, 15 differentially expressed proteins associated with the therapeutic effects of YCL were identified. The functions of these proteins were related to immunity, inflammation, coagulation and hemostasis, oxidation and antioxidation, and lipid metabolism and transport. This study enhanced our understanding on the effective mechanism of YCL in hyperlipidemia.

## Supplementary Material

Supplementary Table 1 desciribes the differentially expressed proteins in detail. It contains “the total proteins”, “proteins with a C.V of less than 0.5”, “proteins upregulated by more than 1.3-fold”, “proteins downregulated by less than 0.769-fold”, “proteins upregulated by more than 1.5-fold”, and “proteins downregulated by less than 0.667-fold”.

## Figures and Tables

**Table 1:** Effect of YCL on serum lipid profile in hyperlipidemic rats ($\overset{-}{x}\pm s$, *n* = 10).

Groups	TC (mmol/L)	TG (mmol/L)	HDL-C (mmol/L)	LDL-C (mmol/L)
Control	1.568 ± 0.049	0.615 ± 0.087	1.584 ± 0.069	0.949 ± 0.057
Model	2.827 ± 0.051^*∗*^	1.228 ± 0.073^*∗*^	0.640 ± 0.066^*∗*^	1.882 ± 0.074^*∗*^
YCL	2.109 ± 0.059^Δ^	0.975 ± 0.054^Δ^	1.148 ± 0.056^Δ^	1.180 ± 0.070^Δ^
XZK	2.123 ± 0.061^Δ^	1.023 ± 0.074^Δ^	1.117 ± 0.063^Δ^	1.206 ± 0.046^Δ^

^*∗*^ *P* < 0.05, versus control group. ^Δ^*P* < 0.05, versus model group.

**Table 2:** Comparison of MDA, ET-1, and CGRP, among different groups ($\overset{-}{x}\pm s$, *n* = 6).

Groups	MDA (nmol/L)	ET-1 (pg/mL)	CGRP (pg/mL)
Control	4.624 ± 0.041	3.989 ± 0.064	35.997 ± 1.053
Model	13.698 ± 0.086^*∗*^	9.006 ± 0.056^*∗*^	24.925 ± 1.195^*∗*^
YCL	9.992 ± 0.072^▲^	4.609 ± 0.061^▲^	31.297 ± 0.877^▲^
XZK	10.071 ± 0.065^▲^	4.670 ± 0.048^▲^	30.842 ± 0.991^▲^

^*∗*^ *P* < 0.05, versus control group.  ^▲^*P* < 0.05, versus model group.

**Table 3:** Pathway enrichment analysis of differential expression of proteins.

Pathway	Number of proteins	Pathway ID
Systemic lupus erythematosus	20	rno05322
Complement and coagulation cascades	19	rno04610
Metabolic pathways	16	rno01100
Alcoholism	14	rno05034
Viral carcinogenesis	13	rno05203
*Staphylococcus aureus* infection	10	rno05150
Malaria	6	rno05144
African trypanosomiasis	6	rno05143
Platelet activation	6	rno04611

**Table 4:** Significant differential expressions of proteins in YCL-treated rats and the model rats.

Accession^a^	Sequence coverage (%)^b^	Peptides (95%)	Gene name	Protein name^c^	115 : 113^d^	119 : 113^d^	115 : 116^d^	119 : 116^d^
*Immune*								
M0RBF1	71.26	126	C3	Complement C3	0.488	0.296	0.597	0.363
P08649	38.51	46	C4	Complement C4	0.421	0.360	0.560	0.483
G3V9R2	52.15	56	Cfh	Protein Cfh	0.575	0.402	0.766	0.535
P55314	39.73	16	C8b	Complement component C8 beta chain	0.497	0.334	0.718	0.479
B0BNN4	33.12	8	Cfp	Complement factor properdin	0.692	0.511	0.832	0.619
Q811M5	22.81	8	C6	Complement component C6	0.550	0.466	0.895	0.766
D3ZPI8	61.59	7	C8g	Complement component 8, gamma polypeptide (predicted), isoform CRA_a	0.685	0.698	0.637	0.655

*Lipid metabolism and transport*								
Q5I0M1	65.22	17	ApoH	Apolipoprotein H	0.520	0.445	0.530	0.461

*Inflammation *								
P11762	93.33	48	Lgals1	Galectin-1	0.525	0.711	0.417	0.565
Q8K3R4	48.36	12	Adipoq	30 kDa adipocyte complement-related protein	2.168	1.854	1.786	2.089
A0A0G2K4G5	21.41	12	Fndc1	Fibronectin type III domain-containing protein 1	0.425	0.525	0.780	0.946

*Coagulation and hemostatic processes *								
P14480	73.28	26	Fgb	Fibrinogen beta chain	0.555	0.433	0.832	0.649
P02680	54.83	20	Fgg	Fibrinogen gamma chain	0.457	0.328	0.711	0.511
P08934	30.99	10	Kng1	Kininogen-1	0.655	0.625	0.667	0.631

*Oxidation and antioxidation*								
B6DYP8	72.40	20	Gsta1	Glutathione S-transferase	2.270	1.247	2.249	1.236

^a^Database accession numbers; ^b^percent sequence coverage of significantly differentially expressed proteins; ^c^name and categories of the proteins identified; ^d^ratios of treatments/models.
